# Heated Debates: Hot-Water Immersion or Ice Packs as First Aid for Cnidarian Envenomations?

**DOI:** 10.3390/toxins8040097

**Published:** 2016-04-01

**Authors:** Christie L. Wilcox, Angel A. Yanagihara

**Affiliations:** 1 Department of Tropical Medicine, Medical Microbiology and Pharmacology, John A. Burns School of Medicine, University of Hawaiʻi at Mānoa, Honolulu, HI 96822, USA; 2 Békésy Laboratory of Neurobiology, Pacific Biosciences Research Center, School of Ocean and Earth Science and Technology, University of Hawaiʻi at Mānoa, Honolulu, HI 96822, USA; ayanagih@hawaii.edu

**Keywords:** jellyfish, venom, sting, first aid, Cubozoa, Scyphozoa, Hydrozoa, hot-water immersion, ice packs

## Abstract

Cnidarian envenomations are an important public health problem, responsible for more deaths than shark attacks annually. For this reason, optimization of first-aid care is essential. According to the published literature, cnidarian venoms and toxins are heat labile at temperatures safe for human application, which supports the use of hot-water immersion of the sting area(s). However, ice packs are often recommended and used by emergency personnel. After conducting a systematic review of the evidence for the use of heat or ice in the treatment of cnidarian envenomations, we conclude that the majority of studies to date support the use of hot-water immersion for pain relief and improved health outcomes.

## 1. Introduction

According to some estimates, more than 150 million cnidarian envenomations occur worldwide every year [1]. Adverse reactions range from minor local pain to severe and life-threatening cardiovascular collapse, and can vary depending on the age and health status of the victim, the amount of tentacle contact, and the species involved in the envenomation. However, despite the frequency of cnidarian envenomations and the medical urgency, there is much debate and disagreement between national and international guidelines as to the most effective first-aid measures to reduce pain or pathology from cnidarian stings. Conflicting recommendations confuse medical professionals and the general public and undermine the ability of on-site first responders to provide the best care. 

Immersion in hot water (~45 °C) is a well documented and commonly recommended therapy for many kinds of marine envenomations, including the painful stings from echinoderms and venomous fish [2]. The mechanism of action is not entirely clear, but there is evidence that marine venoms are heat labile, and thus immersion of the sting area in hot water is thought to inactivate key venom components. Others have postulated that the temperatures needed for such inactivation are much higher (~60 °C), and thus suggest that the pain relief which occurs during hot-water immersion instead results from physiological reactions to heat application (such as increased subcutaneous blood flow). 

Conversely, it was once thought that cryotherapy or immersing envenomed areas in ice water might slow the movement of venom towards the core (heart), and thus might lead to better outcomes in deadly envenomations. Experimental evidence does not support the use of cryotherapy or cold-water immersion in reducing pathology or improving clinical outcomes, however, and the practice is no longer recommended [3]. Ice packs are still commonly used in non-life-threatening arthropod envenomations, such as bee stings. In such cases, there is no presumed action on the venom, and rather, relief is believed to occur through the anti-inflammatory effects of cold.

Hot water and ice are both inexpensive and readily available in many locations where cnidarian stings might occur, and thus would make ideal sting treatments if effective. Thus, the purpose of this review is to examine the evidence for the application of either cold (ice) or heat (hot-water immersion or hot showers) in the treatment of cnidarian envenomations. We performed a systematic review of published research to date (as of 25 January, 2016) by searching the following electronic databases: MEDLINE, CINAHL, Cochrane Reviews, and Google Scholar. Search terms were: jellyfish or cubozoan or cnidaria or physalia or ”Portuguese man-o-war” or “sea nettle” and sting or envenomation or pain or treatment or heat or “hot-water immersion” or ice. We also examined reference lists from studies and guidelines discovered in our searches for potentially relevant papers missed in our database searches. More than 2000 articles were found via these searches, which were carefully examined and narrowed to the 19 relevant publications and abstracts discussed in Levels I to IV in this paper (not including published abstracts or the additional six case series without heat or cold application which are also discussed in Level III).

### Levels of Evidence

The Levels of Evidence used in this review were adapted from the US Agency for Health Care Policy and Research Classification, similar to that reported elsewhere [2,4]. They are as follows:

- Evidence from randomized controlled trials
  - Meta-analysis of randomized controlled trials
  - Evidence from at least one randomized controlled trial
- Evidence from non-randomized controlled trials
  - Quasi-randomized controlled trials
  - Non-randomized controlled trials
- Evidence from non-experimental observational studies
  - Case series with stastistical comparison of clinical outcomes
  - Retrospective case series
  - Prospective case series
- Evidence from other sources
  - Controlled bench studies or basic research
  - Uncontrolled studies
  - Expert letters or opinions
  - Systematic reviews

## 2. Level I Evidence: Randomized Controlled/Paired Trials

Three studies were determined to fit the criteria for Level I evidence. However, it is important to highlight the difficulty of obtaining a high-quality randomized trial for the treatment of cnidarian envenomations, particularly when temperature is one of the test conditions. How much tentacle contact/venom delivery occurred, the duration of time between the sting event and when medical care is sought (and thus when the victim is enrolled into the trial), and what measures are performed during that time period (e.g., if the victim rinses the sting site in fresh water, or applies their own remedies prior to approaching a life guard) are all variables that may have a significant impact on clinical outcomes after a sting event and are difficult or impossible to control. Furthermore, there is no adequate way to blind the study participants or administrators when the different treatments are temperature based. That said, summaries of three trials are presented in Table 1.

Two of the Level I studies examined stings from the pan-tropical cubozoan *Alatina alata* (synonymous with *A. moseri*, reported as *Carybdea alata*). Responses to stings from this large box jellyfish can range from mild to severe, including life-threatening Irukandji syndrome. Thomas *et al.* [5] evaluated the short-term effects of hot or cold pack application as compared to a placebo (less than 15 min post treatment) in 133 swimmers. All participants received vinegar dousing prior to hot or cold pack application. Hot pack application reduced pain scores at 5 and 10 min post treatment, and there was a significantly higher odds ratio (5.2) for the complete cessation of pain with hot pack application compared with placebo. Hot pack application also reduced pain scores when compared directly with cold pack application at 10 min. However, the authors themselves noted that the effect only bordered on clinical significance. In a randomized paired trial on 25 healthy volunteers, Nomura *et al.* [6] compared hot-water immersion with other common first-aid measures (papain and vinegar). Volunteers were stung on each arm, and one was submerged in hot water and the other received either vinegar or papain. Pain scores were lower with hot-water immersion at 4 and 20 min. However, the use of potential active substances rather than a placebo may have affected the control baseline, and the distraction of two simultaneous stings has been noted as a potential confounding factor [2,3]. 

The third Level I study was a randomized controlled trial to test the efficacy of hot-water immersion with ice packs for pain relief from *Physalia* stings (class Hydrozoa). Loten *et al.* [7] compared the pain reported from 96 sting victims which were randomized to receive either hot-water immersion (49 subjects) or ice packs (47 subjects). A significantly larger percentage of the hot-water group reported less pain at 10 and 20 min of treatment (53% and 87%, respectively). At the halfway interim analysis, the trial was halted as hot-water immersion was determined to be more effective (*p* = 0.002). The hot-water group did begin the study with a higher degree of initial pain reported, though, which may indicate allocation bias. In addition, only severe stings were admitted into the study, which may also have influenced the results.

A fourth trial which would have been included as Level I evidence was reported in an abstract in *Emergency Medicine* in 2002 [8], comparing hot showers to ice packs in *Physalia* envenomations off the coast of Sydney, Australia in a randomized crossover trial. The abstract stated that 54 adults were randomized to receive hot showers or cold pack application (27 each), while only 24 completed crossover. Hot showers reduced total treatment time (11.0 min *versus* 14.5 min) and led to greater percentage pain reduction (82.1% *versus* 65.6%) according to the combined results, with 48% of hot-shower patients reporting complete pain cessation (while only 29% reported cessation with ice packs). However, this study lacked follow up, and to our knowledge, complete methods and results were never published in a full, peer-reviewed paper, thus they are not included in Table 1.

## 3. Level II Evidence: Non-Randomized Controlled Clinical Trials

No Level II evidence for the use of hot-water immersion or ice packs for cnidarian envenomations was found. One study, which would have been included as Level II evidence, was reported in an abstract in the *Journal of Toxicology Clinical Toxicology* in 2000 [9] and compared the use of ice or hot-water immersion in a quasi-randomized trial. Adherent tentacles removed with vinegar; on odd days, sting area was immersed in hot water (110 °F/ 43 °C), while on even days, ice packs were applied. Follow up calls at 1, 4, and 24 h. Twenty-four patients completed the trial without protocol violations; 16/16 hot water and 5/8 ice reported pain relief within 60 min. Three patients failed to obtain pain relief with ice packs and subsequently experienced pain relief with immersion in hot water. Hot water was reported as significantly better than ice at relieving pain (*p* < 0.05). However, this study was never published as a full, peer-reviewed paper, thus it is not included as Level II evidence. 

## 4. Level III Evidence: Retrospective Case Series

Three retrospective case series were identified where heat application or ice packs were used. The results of these studies are summarized in Table 2. Peca *et al.* [10] examined 40 cases of *Carybdea marsupialis* envenomation in individuals admitted to the Accident and Emergency Department of San Giorgio Hospital in Cervia, Italy, in July and August, 1994. No systemic symptomology was found in any of the patients, but all had linear erythematous weals ranging in length from a few centimeters up to 20 centimeters. In eight patients, the sting area was soaked in “warm salty water to deactivate the toxins, which are thermolabile, until resolution of symptoms was obtained, a result achieved in all patients in about 30 min”. No statistical analyses were performed, and the temperature of the water was not noted.

Yoshimoto and Yanagihara [11] examined the outcomes of 177 definite or probable cnidarian stings over a five-year period in Hawaiʻi (1994–1998). Of the 177, 60 were treated with heat in some form (53 with hot showers, seven with localized hot packs), but only 42% of cases contained sufficent outcome information to be analyzed. Heat was found to be superior to parenteral analgesics (odds ratio of 11.5, *p* = 0.08), particularly if analyses were restricted to hot showers (odds ratio of 22.0, *p* = 0.0485). Of the 25 cases treated with heat and in whom outcome information was available, 23 reported relief of pain. When only patients presenting with symtoms of Irukandji syndrome were considered, heat still appeared to be superior to analgesics and benzodiazepines. Heat application was not reported to be deleterious in any of the 60 cases. 

Currie *et al.* [12] examined patients presenting with jellyfish stings at the Royal Darwin Hospital and remote coastal community health clinics in the Northern Territory between 1 April 1991 and 30 May 2004. Ice was applied in 71% of cases, with additional analgesia required almost half (48%) of the time, and parenteral narcotics necessary in almost a third (30%) of cases where ice was used. No statistical analyses were conducted to determine if the application of ice affected clinical outcomes.

In addition, there are six additional case series of cnidarian envenomations, in which neither heat nor ice was applied. Maretić and Russell [13] discussed the outcomes of 55 cases of *Anemonia sulcata* envenomations from the Adriatic Sea, noting that, for many of the cases, no immediate treatment was given or the treatment was ineffective; vinegar, wine, urine, manure, mustard, ammonia, alcohol, and petrol as common local measures, though the most commonly employed measure was “rubbing a fig over the injured area”. No life-threatening cases were reported.

Labadie *et al.* [14] reported the results of 1079 *Physalia physalis* stings in patients, who reported to a local poison center in Bordeaux, France from 2008 to 2011. Systemic symptoms were reported in 20% of cases, including gastrointestinal, cardio-respiratory and neurological symptoms. In 2011, 8% of patients presented with severe enough systemic manifestations (such as respiratory distress) to be considered life-threatening by the attending physician, though no fatal cases were reported. The authors noted that hot-water immersion was not proposed as the evidence for its efficacy was for a different species (*Physalia utriculus*), and the authorities deemed hot-water immersion “too difficult to perform in case of collective accident with numerous patients to manage at the same time”.

Four of the additional case series examined patients presenting with Irukandji syndrome; none of cases received ice packs or heat treatments for either the initial sting pathology or systemic symptoms. Little and Mulcahy [15] identified 62 cases of Irukandji syndrome from Cairns emergency departments in 1996. Of these, 61% received opiate analgesia, and 81% had vinegar applied, which appeared to have no adverse effect. Huynh *et al.* [16] noted that, of 128 patients who were discharged with a diagnosis of “marine stings” from Cairns Base Hospital in Queensland, Australalia, between June 2001 and July 2002, 116 had Irukandji syndrome symptoms. These cases required an average dose of 31 mg of morphine; 36% were discharged directly from the emergency department within eight hours of presentation and 47% were discharged from the emergency department observation ward the next day. Macrokanis *et al.* [17] found that, of 111 patients with marine stings between January 2001 and July 2003, 88 had symptoms of Irukandji syndrome; 38% of those had vinegar applied before admission to the hospital; 50% were hypertensive upon arrival; and 90% required opiate analgesia. Nickson *et al.* [18] described the outcomes of 87 Irukandji cases from Australia’s Northern Territory between 1990 and 2007. Systemic features were common, including hypertension and electrocardiographic abnormalities, but no fatalities occurred. Vinegar was used in the majority of cases.

## 5. Level IV Evidence: Controlled Bench Studies, Expert Opinions/Letters, and Systematic Reviews

Thirteen studies were determined to fit the criteria for Level IV evidence: 1 controlled bench study, three systematic reviews, two uncontrolled studies, and seven expert opinions, which are summarized in Table 3.

### 5.1. Controlled Bench Studies

Yanagihara *et al.* [19] developed a novel array of *ex vivo* cnidarian envenomations models with which to test various first-aid measures. In the most sophisticated human tissue model, red blood cells were suspended in an optically clear, low-melting point agarose protected by a skin substitute made from sterilized porcine small intestine. Live tentacles were applied to the skin and allowed to sting spontaneously for 5 min, at which point they were removed and a pack containing either ice water or hot water (45 °C) was applied for 5 min. Size of the lytic zone was calculated after one hour of incubation at 37 °C and compared to controls which received neither treatment. Hot-water treatment significantly reduced the size of the lytic zone (*p* < 0.05), while ice water had no significant effect. 

### 5.2. Systematic Reviews

Three systematic reviews were identified in our search. The first examined evidence for or against hot-water immersion for all marine envenomations [2], finding that “Current published evidence seems to support the use of hot-water immersion in the treatment of non-life threatening marine envenomation, alongside other established first aid measures.” Similarly, a systematic review six years later also concluded that the experimental evidence supports the use of heat to inactivate venom components [4], and a similar systematic review for the Cochrane Database found limited support for the use of hot-water immersion and no support for the use of ice to treat cnidarian envenomations [20].

### 5.3. Uncontrolled Trials and Expert Opinions/Letters

Exton *et al.* [21] provided the first evidence in support of the use of ice in an uncontrolled trial. *Physalia* stings were classified by severity (severe, moderate or mild) and pain severety (severe, moderate or mild) for 143 sting victims at the beach. For all cases, ice packs were applied for 5–10 min, after which pain was assessed. If pain persisted, an ice pack was applied for an additional 5–10 min, then pain was reassessed. All of the mild pain cases resolved after one ice pack application, while about a quarter of the moderate and over a third of the severe cases required additional treatment. Pain persisted after two ice packs in 25% of the severe cases and 2% of the moderate ones. While ice was credited with symptom relief, there was no placebo or control performed. Thus, it is unclear how many cases would have resolved spontaneously without ice.

Taylor [22] performed a quick experiment using a local *Carybdea* species at a doctor’s seminar at the Busselton Hospital in Western Australia, which was published as a letter to the editor. Five volunteers (doctors and medical students) were stung by dragging tentacles over the forearm, two stings per arm for a total of four stings. Each volunteer then received four treatments, one at each sting site: ice, vinegar, aluminium sulfate, and hot water (approximately 45 °C). Hot water led to 85% pain relief during treatment and 88% continuing pain relief after treatment, while ice relieved pain in 25% of cases during treatment and relief was continued in only 2% of cases after treatment. The simultaneous testing of four treatments may have made pain judgements difficult, however, and no statistics were performed. 

Several experts have described personal successes with ice or heat, or warned of potential complications. The earliest examples found of cold water or ice as a potential treatment contraindicated their use, particularly for *Physalia*. Bennett [23] found cold-water application increased the symptoms of his own *Physalia* sting in 1834, while Barnes [24] noted that in the case of one sting, ice “re-awakened” pain symptoms when applied more than four hours after the sting, while warm water had no effects.

Burnett [25] appears to be the first to recommend against the use of heat, citing a single example where visible erythematous lymphangitic spread on the extremity of an adult male who had immersed his arm in a 31 °C water bath for 13 min following a *Physalia* sting. Burnett thus concluded that “the use of local heat is contraindicated since it increases the permeability of the venom”. However, the temperature of the water used was well below body temperature and thus was not “hot water”, nor was it in the known range of venom inactivation (somewhere between 40–60 °C, Table 4). Burnett also noted that cold compresses were not effective, as any pain reduction which occurs while the compress is applied “returns once the extremity is rewarmed”. He later softened his position on both [30], writing that “additional pain relief can be achieved by cold packs or warm compresses,” with the caveats that “care must be taken not to over-chill a small patient nor to apply sufficient heat that vasodilatation opens extra avenues for venom entry to the core of the body”. However, fears of negative outcomes from vasodilation due to heat have not been corroborated by the clinical literature (see evidence in Levels I–III; Table 1, Table 2 and Table 3). Other opinions supportive of ice packs for pain cite unpublished data [26] or opinions of others who cite unpubished data [28], showing how conclusions lacking scientific support are propagate through the literature.

Taylor [27] reported on four stings from the cubozoan *Tamoya gigantua*, two of which were his own. For the first three cases, no treatment steps are described. In the last case, the author was stung extensively on the hand and head while snorkeling, and thought to test the efficacy of hot-water immersion by soaking his hand and head in water “as hot as he could stand”. Hot water instantly relieved pain, but reliefwas transient if the area was removed before 20 min of treatment. Hot towels and a hot shower were not effective. 

More recently, Little [29] reviewed many of the studies included in the first three levels of evidence in this paper, and concluded that “there is very little evidence to support the use of ice packs for jellyfish stings, and there is increasing evidence that hot-water immersion is more effective in reducing the pain of jellyfish stings” He also had strong words for the Australian Resucitation Council (ARC) and others who write clinical guidelines, stating that, “It is time that bodies, such as the ARC, recommend the first-aid treatment supported by the evidence”.

## 6. Mechanism of Action of Heat/Hot-Water Immersion 

The prevailing hypothesis as to the mechanism by which heat treats cnidarian envenomations is that the temperatures used inactivate venom components. There is abundant evidence from *in vitro* and *in vivo* studies which suggest that cndarian venoms from all classes in the phylum are heat labile (Table 4) [31,32,33,34,35,36,37,38,39,40,41,42,43,44,45]. Some have suggested that the mechanism by which heat provides pain relief is unrelated to inactivation of venom components. The alternative explanation proposed is that hot-water immersion has a direct modulatory effect on pain receptors leading to a reduction in perceived pain [46]. 

## 7. Hot-Water Immersion for Other Envenomations

Hot-water immersion is considered the standard of care for most marine and freshwater envenomations (Table 5) [47,48,49,50,51,52,53,54,55,56,57,58,59,60,61,62,63,64,65,66,67,68,69,70,71,72,73]. It is the primary recommendation for stings from echinoderms (incluing sea stars, sea urchins, and sea cucumbers) [47,48,49,50,52,53,54,55,57,59,60], as well marine and freshwater fishes (including sting rays, scorpionfishes, weeverfishes and catfishes) [47,48,49,50,51,52,53,54,55,56,57,61,62,63,64,65,66,67,68,69,70,71,72,73]. Hot water has also been recommended for stings from polychete worms [58]. These injuries are generally considered mild to moderate, and not life-threatening. However, hot-water immersion has been recommended for the potentially lethal stings from stonefishes [47,48,49,50,51,52,53,54,55,56,57,71,72,73] and, on some occasions, cone snails [47,50]. The only marine envenomations where hot-water immersion has not been recommended are bites from sea snakes, which should be treated in the same manner as other neurotoxic snake envenomations, including antivenom administration. 

For terrestrial envenomations, hot-water immersion was less commonly recommended (Table 5) [74,75,76,77,78], although there has been some support for the use of targeted, localized heat in the treatment of insect bites and stings [79]. Heat therapy is specifically contraindicated for recluse spider envenomations (*Loxoceles* spp.), as the dermonecrotic venom factor sphingomyelinase D increases its activity as temperature increases [75]. This group of spiders are the only ones with this particular enzyme, however, so it is unclear if hot-water immersion would be appropriate in other spider envenomations. Preliminary case reports and investigations suggest heat application may aid in pain relief from scorpion stings [76,77] and centipede bites [78]. A prospective cohort study (*N* = 146) from Germany found that the targeted application of heat (maximum of 51 °C) for 3–6 s using the Bite Away^®^ device (RIEMSER Pharma GmbH, Greifswald, Germany) led to a reduction visual analog scale scores for pain, swelling and pruritus from mosquito bites and bee and wasp stings [79]. Neither heat application nor ice are recommended for snakebites [80], though we could find no clinical documentation of the use of hot-water immersion. Cohen *et al.* [81] found that, in a mouse model, hot-water immersion caused increased mortality in one of the two experiements performed with the hemotoxic venom of *Agkistrodon piscivorus*, contraindicating the use of hot-water immersion for snakebites. Interestingly, they also noted using heat application in their own clinical pathway for snakebite; however, they stated that heat is only applied after antivenom has been administered and provided no information about the clinical efficacy of heat application.

## 8. Conclusions

The preponderance of evidence demonstrates that hot-water immersion is a safe and effective method of reducing pain from cnidarian envenomations, and is also associated with improved clinical outcomes. No studies or cases were found where hot-water immersion led to worsened symptoms or poorer clinical outcomes. Fears of negative effects of immersing a stung limb in 45 °C water for 20 min are not supported in the literature; this is not surprising, given that hot-water immersion is considered safe and is recommended for the treatment of stonefish stings and other life-threatening marine envenomations with potential cardiovascular complications.

There are two noted caveats to the use of hot-water immersion in the case of envenomations: temperature safety and temperature maintenance. While there is considerable concern regarding thermal injuries from hot-water immersion, there was only a single recorded case of significant thermal burn from over 200 cases of the use of hot-water immersion reviewed by Atkinson *et al.* [2]. Similarly, the difficulty of maintaining hot-water at an appropriate temperature on site can be overcome using several methods, including the use of reusable hot packs instead of water, continual hot-water input (such as from a shower or hose at the correct temperature), or an immersion container with better insulatory capabilities. To that end, Lau *et al.* [54] tested the use of different containers on temperature maintenance, finding that thermal insulators (standard coolers) were able to maintain water temperature effectively for a full 30 min. 

## Figures and Tables

**Table 1:** Published Level I evidence (randomized controlled/paired trials) of either ice or hot-water immersion for the treatment of cnidarian stings.

Study	Type	*N*	Species	Outcome	Comments
Thomas *et al.* 2001 [5]	Randomized controlled trial	133	*Alatina alata* (Cubozoa)	Cessation of Pain Odds Ratio (OR) and Percentage: Hot pack: 5.2 (1.3 to 22.8), 41% Cold pack: 0.5 (0.1 to 2.1), 33% Placebo: 1.0, 29% Pain Scores at 0, 5, 10 min: Hot pack *vs.* cold pack: 42.3 ,31.3, 27.5 * *vs.* 38.3, 32.8 *, 36.2 Cold pack *vs.* placebo: 38.3, 32.8 *, 36.2 *vs.* 38.6, 37.7, 38.2 Hot pack *vs.* placebo: 42.3, 31.3 *, 27.5 * *vs.* 38.6, 37.7, 38.2	Inadequate blinding, not analyzed on intent to treat basis, poor randomization technique, altered cessation of pain definition after start of trial.
Nomura *et al.* 2002 [6]	Randomized paired trial	25 pairs of stings	*Alatina alata* (Cubozoa)	Pain scores at 0, 4, 20 min: Hot-water immersion: 3.6, 2.1 **, 0.2 ** Control: 3.7, 3.2, 1.8	Controls potentially active (vinegar/papain used rather than placebo); dual sting may have made it difficult for participants to evaluate
Loten *et al.* 2006 [7]	Randomized controlled trial	96	*Physalia* spp. (Hydrozoa)	Percentage with reduced pain at 10, 20 min: Hot-water immersion: 53% *, 87% ** Ice pack: 32%, 33%	Inclusion of only severe stings and possible allocation bias (baseline differences in severity)

Notes: * Indicates *p* < 0.05; ** Indicates *p* < 0.001.

**Table 2:** Published Level III evidence (case series) of either ice or hot-water immersion for the treatment of cnidarian stings.

Study	Type	*N*	Species	Outcome	Comments
Peca *et al.* 1997 [10]	Retrospective case series	40 cases, 8 patients received soak in “warm salty water”	*Carybdea marsupialis* (Cubozoa)	No systemic symptomology, all had linear erythematous weals ranging in length from a few centimeters up to 20 centimeters. Patients that received warm water soak had symptoms resolve in 30 min or less	No statistical analyses were performed; temperature of the water was not noted.
Yoshimoto & Yanagihara 2002 [11]	Retrospective case series	177 cases; 60 received heat (53 with hot showers, 7 with localized hot packs; outcomes available for only 25)	*Alatina alata* (Cubozoa)	Twenty-three/Twenty-five cases with heat reported relief of pain OR heat *vs.* parenteral analgesics: 11.5 OR hot showers *vs.* parenteral analgesics: 22.0* Heat also superior to analgesics and benzodiazepines in cases with Irukandji symptions	Temperature of water was not noted.
Currie & Jacups 2005 [12]	Retrospective case series	606 cases; 225 from *C. fleckeri*	*Chironex fleckeri* and other box jellies (Cubozoa)	Ice applied in 71% of cases; analgesia in addition to ice was required in 48% of cases, with parenteral narcotic required in 30%. One fatal case occurred in a 3-year-old with 1.2 m of visible tentacle (cardiopulmonary arrest within minutes of the sting)	No statistical analyses were performed to determine if ice improved outcomes.

Notes: OR = Odds Ratio; * Indicates *p* < 0.05.

**Table 3:** Published Level IV evidence (bench studies, uncontrolled trials, expert opinions, systematic reivews) of ice or hot-water immersion for the treatment of cnidarian stings.

Study	Type	Species	Description	Outcome
Yanagihara *et al.* 2016 [19]	Controlled bench study	*Alatina alata* (Cubozoa)	*Ex vivo* model used to evaluate potential treatments. Model consisted of live human red blood cells suspended in an optically clear agarose and protected by a skin substitute made from sterlized porcine instestine (*n* = 3 each for hot-water pack, ice pack and no treatment).	Use of hot-water pack abolished hemolysis; ice pack had no significant effect.
Atkinson *et al.* 2006 [2]	Systematic Review	All venomous marine species	Electronic databases searched: MEDLINE, EMBASE, and CINAHL as well as The Cochrane Library, the *Britich National Formulary*, and Toxbase for guidlelines, and Google, and manual search of reference lists. Search strategy: ((hot water or heat).mp. or exp *Heat/) and (exp *Fishes/or exp *“Bites and Stings”/or fish sting.mp. or exp *Fishes, Poisonous/or exp *Fish Venoms/or exp *Jellyfish/or envenomation.mp.).	“Current published evidence seems to support the use of hot-water immersion in the treatment of non-life threatening marine envenomation, alongside other established first aid measures.”
Ward *et al.* 2012 [4]	Systematic Review	Cnidarians found in North America and Hawaiʻi	Electronic databases searched: MEDLINE, EMBASE, CINAHL, Cochrane Reviews, and Google Scholar, and manual search of reference lists. Search terms: “jellyfish AND envenomation,” “jellyfish AND sting,” “jellyfish AND treatment,” “jellyfish AND nematocyst AND discharge,” and (jellyfish OR cubozoan OR cnidaria OR physalia OR ”Portuguese man-o-war” OR “sea nettle”) AND (pain OR treatment OR vinegar OR ammonia OR heat OR water OR nematocyst).	“Experimental evidence for North American and Hawaiian species supports [heat inactivation of venom] because hot-water immersion reduces pain from *Carybdea alata* stings as well as *Physalia physalis*. Cold packs, however, likely do not significantly alleviate pain… Hot water and topical lidocaine appear more universally beneficial in improving pain symptoms and are preferentially recommended.”
Li *et al.* 2013 [20]	Systematic Review	All Cnidarians	Electronic databases searched: MEDLINE, EMBASE through Ovid SP, Web of Science (all databases 1899 to 21 October, 2013), and CENTRAL (1980 to October 2012, with a repeated search in 2013), as well as a manual search of reference lists, guidelines, and the World Health Organization International Clinical Trials Registry Platform; No language, publication date, or publication status restrictions.	”It is still unclear what type of application, temperature, duration of treatment and type of water (salt or fresh) constitute the most effective treatment.”
Exton *et al.* 1988 [21]	Uncontrolled Trial	*Physalia* sp. (Hydrozoa)	Stings classified by severity (severe, moderate or mild) and pain severety (severe, moderate or mild); ice packs then applied for 5–10 min, after which pain was assessed. If pain persisted, ice was applied for a further 5–10 min.	*N* = 143; Pain relief after 1 or 2 ice packs: Severe pain: 62.5%, 75% Moderate pain: 77%, 98% Mild pain: 100%
Taylor 2007 [22]	Uncontrolled Trial/Expert Letter	*Carybdea* sp. (Cubozoa)	Five volunteers (doctors and medical students) stung by dragging tentacles over forearm, with two stings per arm. Each volunteer then received four treatments, one at each sting site: ice, vinegar, aluminium sulfate, and hot water	Hot water was “the only successful treatment, relieving 88% of the pain”; pain relief obtained in 4–10 min, while other treatments “incomplete and temporary.”
Bennett 1834 [23]	Expert Opinion/Case History	*Physalia* sp. (Hydrozoa)	The author captured a specimen and allowed himself to be stung out of curiosity. He described a “violent aching pain”, which he attempted to relieve with cold water.	Cold water applied to the sting site “was found to rather increase than dimish the effects.”
Cleland & Southcott 1965 [24]	Expert Opinion/Case History	*Physalia* sp. (Hydrozoa)	Case notes from Jack Barnes quoted by authors; Case described as the *Physalia* sting on the arm of a 21-year-old male. Site was treated by rubbing dry sand and, later, a weak ammonia solution, neither of which seemed to relieve pain; pain spontaneously resolved about 20 min after sting. About 1 hr later, returning to the water led to immediate pain similar to initial sting.	Pain was also “reawakened” 4.5 h after the sting by rubbing the area with ice, producing an “electric feeling,” while warm water had no effects.
Burnett 1989 [25]	Expert Opinion/Case History	*Physalia* sp.(Hydrozoa)	The author summarizes his experience and recommendations for the treatment of jellyfish stings, citing one particular case where visible erythematous lymphangitic spread could be seen on the extremity of an adult male who had immersed his arm in a 31 °С water bath for 13 min following a *Physalia* sting.	“The use of local heat is contraindicated since it increases the permeability of the venom. … Cold compresses have not been effective against the pain. Pain is reduced as long as cold is applied to the sting site but returns once the extremity is rewarmed.”
Currie 1994 [26]	Expert Opinion	*Chironex fleckeri* (Cubozoa)	Review of research on *C. fleckeri* venom and clinical applications.	States that ice packs provide good pain relief for mild stings, citing the author’s unpublished data.
Taylor 2000 [27]	Expert Opinon/Case History	*Tamoya gigantua* (Cubozoa)	Report of four sting cases, two from the author. For the first three cases, no treatment steps are described. In the last case, the author was stung extensively on the hand and head while snorkeling. He proceeded to apply heat to determine its efficacy.	Hot water instantly relieved pain, but was transient until soaked for 20 min. Hot towels and a hot shower were not effective, so the author soaked his head in a bowl of hot water; a near-complete cessation of pain occured after 20 min.
Tibballs 2006 [28]	Expert Opinion	Australian Cnidaria	Non-systematic literature review and recommendations for treatment of stings from species found in Australian waters.	Cited Currie 1994 as evidence for ice packs; noted hot water efficacious in box jelly stings for species found outside Australia, and that heat inactivation of venom “has been long known”
Little 2008 [29]	Expert Opinion	All Cnidaria	Commentary and review of evidence of ice or heat for cnidarian stings, noting that improvement seen in Exton *et al.* [21] may have been a placebo effect, as suggested by Loten *et al.* [7]. Hot-water immersion, on the other hand, has been tested in multiple studies, which the author notes all suggested hot water was an effective treatment.	“There is very little evidence to support the use of ice packs for jellyfish stings, and there is increasing evidence that hot-water immersion is more effective in reducing the pain of jellyfish stings. It is time that bodies, such as the ARC, recommend the first aid treatment supported by the evidence.”

**Table 4:** Published evidence of heat inactivation of cnidarian venoms or venom components at temperatures below 80 °C.

Study	Species	Methods	Activity Measure	Results
Baxter & Marr 1969 [31]	*Chironex fleckeri* (Cubozoa)	Venom heated to 30, 40, 50, 60, or 70 °C for 10 min Rapid exposure: venom brought “quickly” to 44, 47, 50, 53, 56, or 59 °C and immediately chilled	Lethal activity in mice	10 min exposure: activity lost between 40 and 50 °C Rapid exposure: activity lost at >53 °C
Endean & Henderson 1969 [32]	*Chironex fleckeri* (Cubozoa)	Venom incubated at 37, 42 or 45 °C for 5, 10, 15 or 20 min	Barnacle muscle contraction	Activity lost between 40–42 °C (all activity lost if heated at 42 °C for 5 min, but activity remained after heating at 40 °C for 20 min)
Chung *et al.* 2001 [33]	*Alatina alata* (formerly *Carybdea alata*; Cubozoa)	Venom serially diluted and maintained at 4, 25, 37, 45, 60 or 100 °C for 30 min	Hemolytic activity in sheep red blood cells	Venom potency decreased when exposed to temperatures above 25 °C; activity was “sharply reduced” at 45 °C, and abolished entirely at 100 °C
Monastyrnaya *et al.* 2002 [34]	*Radianthus macrodactylus* (Anthozoa)	Isolated cytolysins heated briefly to 35, 45, 60, and 80 °C	Hemoylytic activity in rabbit or rat red blood cells	Activity decreased linearly with increasing temperature; at 35, 45, 60, and 80 °C caused the loss of 6, 18, 95, and 100% of their initial hemolytic activity.
Carette *et al.* 2002 [35]	*Chironex fleckeri* (Cubozoa)	Venom incubated at 4, 21.5, 33, 39, 43, 48, 53, or 58 °C for 2, 5, or 20 min	Lethal activity in crayfish	Activity decreased overall with increased temperature and duration of exposure. All activity lost at 20 min of 43 °C or 5 min of 50 °C
Koyama *et al.* 2003 [36]	*Chiropsalmus quadrigatus* (Cubozoa)	Venom heated to 40 °C for 10 min	Hemodynamic effects in rabbits	Significant attenuation of activity
Marino *et al.* 2004 [37]	*Aiptasia mutabilis* (Anthozoa)	Venom incubated at 4 °C, 20 °C, 40 °C and 60 °C for 5, 30 or 60 min	Cytotoxicity in monkey Vero cells	All activity lost at 60 °C; activity reduced in a time- and dose-dependent manner at lower temperatures
Noguchi *et al.* 2005 [38]	*Chiropsalmus quadrigatus* (Cubozoa)	Venom heated to 50 °C for 10 min	Lethal and hemodynamic effects in rats	All activity lost
Kang *et al.* 2009 [39]	*Nemopilema nomurai* (Scyphozoa)	A: venom incubated at 4, 20, 40, 60 and 80 °C for 60 min B: venom incubated at 40 °C for 0, 10, 30, 120 or 360 min	Cytotoxicity in H9C2 heart myoblasts	A: all activity lost at temperatures > 60 °C, attenuation of activity in 40 °C incubated venom B: activity reduced in a time- and dose-dependent manner
Feng *et al.* 2010 [40]	*Cyanea nozakii* (Scyphozoa)	Venom incubated at 35, 50, 65 and 80 °C for 20 or 40 min	Lethality in grass carp (*Ctenopharyngodon idellus*)	All activity lost at temperatures > 65 °C; activity reduced in time- and dose-dependent manner
Cuiping *et al.* 2011 [41]	*Cyanea nozakii* (Scyphozoa)	Venom incubated at 20, 40, 60, and 80 °C for 10, 30, or 50 min	Cytotoxicity in H630 cells	Nearly all activity lost at 60 °C for even 10 min; activity lost with increased temperature and incubation time
Pereira & Seymour 2013 [42]	*Chironex fleckeri* (Cubozoa)	Venom incubated at 24, 37, 44, 46, 48, 50, 60, or 100 °C for 20 min	Cytotoxicity in human cardiomyocytes and skeletal myocytes	All activity lost at 44 °C for cardiomyocytes and at 48 °C for skeletal myocytes (80% survival at 44 °C)
Li *et al.* 2013 [43]	*Stomolophus meleagris* (Schyphozoa)	Isolated venom toxin (SmTX) incubated at 4, 15, 25, 37, 45 and 60 °C for 30 min	Hemolysis of chicken red blood cells	Activity decreased with temperature above 37 °C
García-Arredondo *et al.* 2014 [44]	*Millepora complanata* (Hydrozoa)	Venom incubated at 4, 25, 37, 45, 60 and 100 °C for 30 min	Hemolysis of human red blood cells	Activity decreased at temperatures ≥ 45 °C in a dose-dependent manner
Hernández-Matehuala 2015 [45]	*Millepora alcicornis* (Hydrozoa)	Venom incubated at various temperatures between 0 and 100 °C for 60 min	Hemolysis of human red blood cells	Activity sharply decreased when incubated abouve 40 °C, with >50% activity lost at 45 °C

**Table 5:** Evidence and recommendations for the use of heat or hot-water immersion in other venomous animals.

Study	Type	Species	Description
***Marine and Freshwater Envenomations***			
Kizer 1983 [47]	Non-Systematic Review, Expert Opinion	Phyla: Cnidaria, Porifera, Echinodermata, Mollusca, Annelida, and Chordata	Hot-water immersion recommended for envenomations from cnidarians, cone snails, sea urchins, sea stars (noted that may be ineffective for *Acanthaster planci*), sting rays, and venomous fishes.
Aurbach 1991 [48]	Non-Systematic Review, Expert Opinion	Phyla: Cnidaria, Porifera, Echinodermata, Mollusca, Annelida, and Chordata	Hot-water immersion recommended for venomous puncture wounds (fishes, sea stars, and urchins noted); ice packs possible for pain relief from cnidarian stings.
McGoldrick & Marx 1991, 1992 [49,50]	Non-Systematic Review, Expert Opinion	Phyla: Cnidaria, Porifera, Echinodermata, Mollusca, Annelida, and Chordata	Hot-water immersion recommended for envenomations from cone snails, sea urchins, and venomous fishes.
Hawdon & Winkel 1997 [51]	Non-Systematic Review, Expert Opinion	Phyla: Cnidaria, Porifera, Echinodermata, Mollusca, Annelida, and Chordata	Hot-water immersion recommended for envenomations from venomous fish stings.
Fenner 2000 [52]	Non-Systematic Review, Expert Opinion	Phyla: Cnidaria, Porifera, Echinodermata, Mollusca, Annelida, and Chordata	Hot-water immersion recommended for ‘venomous spines’ (fishes and echinoderms), especially stingrays and stonefish. Water temperature recommended at about 43 °C, tested by uninjured party or non-envenomated limb. Ice is recommended for cnidarian stings.
Fenner 2002 [53]	Non-Systematic Review, Expert Opinion	Phyla: Cnidaria, Porifera, Echinodermata, Mollusca, Annelida, and Chordata	Hot-water immersion recommended for “penetrating spines” (venomous fish); ice or cold packs for pain relief from cnidarian envenomations.
Lau *et al.* 2011 [54]	Survey of Clinicians (*N* = 16); Experimental Test of Basin Methods	Phyla: Cnidaria, Chordata (Fishes)	All respondents stated that their patients’ wound pain was relieved after hot-water immersion without major adverse effects. Nine commented on difficulty maintaining temperature. Thermal insulators (e.g., cooler) maintained hot-water temperature effectively, while plastic or metal trays did not.
Ottuso 2013 [55,56]	Non-Systematic Review, Expert Opinion	Phyla: Cnidaria, Porifera, Echinodermata, Mollusca, Annelida, Platyhelminthes and Chordata	Hot-water immersion recommended for envenomations from Cubozoa (Cnidaria), *Physalia* (Cnidaria: Hydrozoa), sea urchins, sea stars, and all venomous fishes. Cold water compress recommended for Schyphozoa (Cnidaria); ice packs for Hydrozoa (Cnidaria).
Reese & Depenbrock 2014 [57]	Non-Systematic Review, Expert Opinion	Phyla: Cnidaria, Porifera, Echinodermata, Mollusca, Annelida, Platyhelminthes and Chordata	Hot-water immersion recommended for envenomations from *Physalia* (Cnidaria), *Chironex fleckeri* (Cnidaria: Cubozoa), sea lillies, brittle stars, sea stars, sea urchins, sting rays, and venomous bonyfishes. Ice specifically contraindicated for sea snakes.
**Phylum Annelida**			
Smith 2002 [58]	Non-Systematic Review, Expert Opinion	Class: Polychaeta	After spine removal, immersion in hot water (110 °F–115 °F) with dilute vinegar or ammonia is recommended for pain relief.
**Phylum Echinodermata**			
Smith 2002 [59]	Non-Systematic Review, Expert Opinion	Classes: Asteroidea, Echinoidea, and Holothuroidea	Conclusion that all echinoderm injuries may involve thermolabile toxins that can be treated effectively with hot water (110 °F–120 °F) immersion or irrigation. Mixture of 1:1 hot water:vinegar is recommended for urchin stings specifically.
Class Echinoidea			
Strauss & MacDonald 1976 [60]	Case Report	Sea Urchin	Twenty-four-year-old diver stung on finger. Presented with extreme pain, which was relieved upon soaking hand in hot water.
**Phylum Chordata**			
Isbister 2001 [61]	Case series; 22 cases	Marine Fishes Classes: Chondrichthyes, Actinopterygii	Stings by stingrays (9), catfish (8), stonefish (1), silver scat (*Selenotoca multifasciata*; 1), unknown (3). All patients presented with severe pain; other symptoms included erythema, 3 cases (14%); swelling, 7 cases (33%); bleeding, 5 cases (24%); numbness, 4 cases (19%); and radiating pain, 3 cases (14%). Mild systemic effects occurred in one case. Hot-water immersion was completely effective in 73% of cases.
Chan *et al.* 2010 [62]	Case series; 33 cases	Marine Fishes Classes: Chondrichthyes, Actinopterygii	Stings by catfish (12), stonefish (7), lionfish (4), stingrays (2), waspfish (2), rabbitfish (2), a silver scat (*Selenotoca multifasciata*; 1), unknown (3). All patients presented with pain at the sting site. Other symptoms included wound swelling, 28 cases, erythema; 13 cases and numbness, 13 cases. Twenty-five received hot-water immersion. No systemic symptoms were noted.
Class Chondrichthyes			
Meyer 1997 [63]	Non-Systematic Review, Expert Opinion	Stingrays Order Myliobatiformes	Hot-water immersion with “water as hot as can be tolderated” for 30–60 min is recommended, even in cases of delayed presentation of symptoms, in addition to removal of foreign bodies as well as antibiotics.
Haddad Jr *et al.* 2004 [64]	Case Series; 84 cases	Freshwater Stingrays Order Myliobatiformes Family: Potamotrygonidae	All cases presented with intense pain, erythema and edema, and almost 81% of cases had systemic manifestations. Hot-water immersion was effective when used (7 cases). Treatment recommendation: hot-water immersion for pain, cleaning of wounds and foreign body removal, tetanus prophylaxis, analgesics if pain not controlled in 2 hrs.
Clark *et al.* 2007 [65]	Case Series; 119 cases	Stingrays Order Myliobatiformes	Of patients that presented within 24 hrs, hot-water immersion relieved pain within 30 min in 88% of cases. There were no adverse effects (such as thermal burns) with this therapy. Antibiotic prophylaxis recommended.
Kamajian *et al.* 2014 [66]	Case Series; 21 cases	Stingrays Order Myliobatiformes	Foot immersed in water, temperature increased every 5 min until pain relieved. Pain relief reported at 115 °F. No additional relief past 118 °F. Suggested: 90 min of 118 °F.
**Phylum Chordata**			
Class Actinopterygii			
Briars & Gordon 1992 [67]	Case Series; 24 cases	Weeverfish Order: Perciformes Family: Trachinidae	All patients treated by immersion of stung limb in hot water for 20 min. Twenty-three of the 24 victims reported less pain.
Kizer *et al.* 1985 [68]	Case Series; 51 cases	Scorpionfishes and Lionfishes Order: Scorpaeniformes Family: Scorpaenidae	Hot-water immersion provided 80% complete relief and 14% moderate relief in 94% of patients.
Haddad Jr *et al.* 2003 [69]	Case Series; 23 cases	Scorpionfishes Order: Scorpaeniformes Family: Scorpaenidae Genus: *Scorpaena*	Patients presented with intense pain, with systemic manifestations in about 70% of cases. Six patients received hot-water immersion, which controlled pain. Other measures of treatment used by the patients themselves, including systemic analgesics (six patients), application of urine at the point of the injury (four patients), alcohol (four patients), and garlic (two patients), had in poor results.
Haddad Jr & Martins 2006 [70]	Case Series; 127 cases	Catfishes Order: Siluriformes Family: Siluridae	Patients presented with intense pain. Many had complications, such as bacterial and fungi infections and retention of bony fragments. Hot-water immersion used in 20% of cases with”excellent” results.
Acott 1990 [71]	Case Report	Stonefish Order: Scorpaeniformes Family: Synanceiidae	Man stung on foot. Presented with pain at site as and in calf and thigh and swelling. Hot-water immersion (~50 °C) provided relief, but pain returned upon removal from water. Three vials of antivenom administered intravenously.
Yamamoto *et al.* 2009 [72]	Case Report	Stonefish Order: Scorpaeniformes Family: Synanceiidae	Thirty-three-year-old man stung on finger. Wound washed and sterilized with alcohol, then hand immersed in hot water. Thirty minutes of hot-water immersion lead to relief of pain and numbness.
Ongkili & Phee-Kheng 2013 [73]	Case Report	Stonefish Order: Scorpaeniformes Family: Synanceiidae	Forty-seven-year-old stung on foot. Given 75 mg intramuscular diclofenac, then 5 mg intravenous morphine with no improvement in pain. Soaked foot in hot water at the highest tolerable point via the elbow skin test. Pain score reduced after 15 min of hot-water immersion and diminished at 1 hr.
***Terrestrial Envenomations***			
**Phylum Arthropoda**			
Haddad Jr *et al.* 2015 [74]	Non-Systematic Review; Expert Opinion	Classes: Arachnida, Diplopoda, Chilopoda, Insecta	Heat application/hot-water immersion is not indicated for any species. Cold compresses are indicated for pain relief in envenomations from centipedes, ants, and caterpillars.
Class Arachnida			
King Jr. & Rees 1985 [75]	Non-Systematic Review; Expert Opinion	*Loxosceles reclusa* Order: Araneae Family: Sicariidae	Heat worsened lesions compared to no treatment. Has an enzyme, sphingomyelinase D, that increases activity in heat. Use cold/ice packs for Brown recluse spider bites.
Isbister *et al.* 2004 [76]	Case Report	Scorpion Order: Scorpiones	Pain relief of scorpion sting when placed in hot coffee, suggesting hot-water immersion may be effective in scorpion stings.
Lobo *et al.* 2011 [77]	Case Report	*Centruroides testaceus* Order: Scorpiones	Six-year-old girl stung in the shoulder. Treated with oral dipyrone and given a hot-water bottle for sting site. Pain relief in <2 h.
Class Chilopoda			
Chaou *et al.* 2009 [78]	Prospective Randomized Trial; *N* = 60	Centipedes Order: Not specified	Treatments were ice packs, hot-water immersion, and analgesia injection. Visual analog score measured before treatment and 15 min after. Ice packs, hot-water immersion, and analgesics are all reduced pain for centipede bites.
Class Insecta			
Müller *et al.* 2011 [79]	Prospective Cohort Study; 146 volunteers	Bees, Wasps, and Mosquitoes Orders: Hymenoptera, Diptera	Of the 146 volunteers, 93 (63.7%) were stung by wasps, 33 (22.6%) were bitten by mosquitoes, and 8 were stung by bees (5.3%). Swelling, pain and prutitus decreased 10 min after the use of the Bite Away^®^ medical device, which non-invasively administers concentrated heat (max of 51 °C) to the skin for a choice of 3 or 6 s.
